# Enhanced disease characterization through multi network functional normalization in fMRI

**DOI:** 10.3389/fnins.2015.00095

**Published:** 2015-03-31

**Authors:** Mustafa S. Çetin, Siddharth Khullar, Eswar Damaraju, Andrew M. Michael, Stefi A. Baum, Vince D. Calhoun

**Affiliations:** ^1^Department of Computer Science, University of New MexicoAlbuquerque, NM, USA; ^2^The Mind Research NetworkAlbuquerque, NM, USA; ^3^Chester F. Carlson Center for Imaging Science, Rochester Institute of TechnologyRochester, NY, USA; ^4^Psychiatry Department, University of New Mexico School of MedicineAlbuquerque, NM, USA; ^5^Electrical and Computer Engineering Department, University of New MexicoAlbuquerque, NM, USA

**Keywords:** fMRI, ICA, spatial normalization, resting state networks, wavelet

## Abstract

Conventionally, structural topology is used for spatial normalization during the pre-processing of fMRI. The co-existence of multiple intrinsic networks which can be detected in the resting brain are well-studied. Also, these networks exhibit temporal and spatial modulation during cognitive task vs. rest which shows the existence of common spatial excitation patterns between these identified networks. Previous work (Khullar et al., 2011) has shown that structural and functional data may not have direct one-to-one correspondence and functional activation patterns in a well-defined structural region can vary across subjects even for a well-defined functional task. The results of this study and the existence of the neural activity patterns in multiple networks motivates us to investigate multiple resting-state networks as a single fusion template for functional normalization for multi groups of subjects. We extend the previous approach (Khullar et al., 2011) by co-registering multi group of subjects (healthy control and schizophrenia patients) and by utilizing multiple resting-state networks (instead of just one) as a single fusion template for functional normalization. In this paper we describe the initial steps toward using multiple resting-state networks as a single fusion template for functional normalization. A simple wavelet-based image fusion approach is presented in order to evaluate the feasibility of combining multiple functional networks. Our results showed improvements in both the significance of group statistics (healthy control and schizophrenia patients) and the spatial extent of activation when a multiple resting-state network applied as a single fusion template for functional normalization after the conventional structural normalization. Also, our results provided evidence that the improvement in significance of group statistics lead to better accuracy results for classification of healthy controls and schizophrenia patients.

## Introduction

Spatial registration of multiple subjects onto a common template is a necessary step while analyzing fMRI data across a group of subjects. This step is required for several reasons: (1) brains differ in shapes and sizes and spatial normalization enforces brain boundaries to overlap to exclude non-brain voxels from the analyses, (2) to ensure that similar anatomical regions are compared across subjects and (3) to label brain regions using a standard predefined coordinates (example MNI space). The above steps are appropriate and critical while analyzing brain images from structural magnetic resonance imaging (sMRI). During the pre-processing of functional MRI (fMRI), conventionally, it is normalized using structural topology and such an initial normalization is needed for the above reasons. In fMRI we measure the blood oxygenation level dependent (BOLD) changes of brain activation and previous work (Mazziotta et al., 2001; Brett et al., 2002) has shown that structural and functional data may not have direct one-to-one correspondence. By this we mean functional activation patterns in a well-defined structural region can vary across subjects even for a well-defined functional task.

Previously (Mazziotta et al., 2001; Brett et al., 2002) discussed the differences in correspondence between the functional and structural landmarks of the human brain. They have pointed out the importance of functional localization and incorporating these differences into algorithms and methods that are applied for realignment and segmentation of neuroimaging data sets. Functionally well-defined areas such as the visual motion or the MT can vary across subjects in terms of size (Watson et al., 1993) or mapped anatomical location (Tootell et al., 1995). Also, Blumensath et al. (2006) introduced a spectral clustering technique for intra-subject parcellation that delineates homogeneous and connected regions and a hierarchical method to derive group parcels. They showed the groups of parcels that well-summarize inter-subject activations. This study was followed by another experiment involving deriving parcellation borders that follow changes in the functional connectivity profile (seed based) at rest state which highly overlap with task state (Blumensath et al., 2013). In recent study (Haxby et al., 2011), hyper-alignment model was introduced by mapping a response-pattern vectors from individual subjects' voxel spaces into the common model space. The results showed that population codes for complex visual stimuli in ventral temporal cortex are common across the individuals. Another study (Hagler et al., 2006) proposed iterative smoothing technique that averages the values of neighboring vertices. Their results provided better spatial uniformity of smoothing when the surface meshes used have large variability in inter-vertex distance. Recently, inter-subject registration algorithm that aligns intra-subject patterns of functional connectivity across subjects was introduced by Conroy et al. (2013). The results showed that the derived alignment by using inter-subject registration algorithm performed successfully. Another study provided by Sabuncu et al. (2010) showed inconsistency of structural anatomical landmarks on the cortex with estimated locations of corresponding functional activity. Results of these studies support our initial thought that incorporation of locally defined functional information may improve the boundaries of these areas and some aspects of within-activation topography at a network level. Therefore, robustly estimation of functional landmarks using resting state fMRI data and using these for functional re-alignment may be an alternative to collecting data from a battery of tasks.

In the past decade, there has been a growing interest to use intrinsic networks (INs) alternatively known as “resting state networks” to investigate the functional organization of the human brain. Previously, large scale of resting state fMRI data has been collected by Biswal et al. (2010) and Allen et al. (2011a). Also, independent component analysis (ICA) has been a very popular tool to analyze resting state fMRI data and investigate functional connectivity (Beckmann et al., 2005; Damoiseaux et al., 2006; Calhoun et al., 2008; Harrison et al., 2008; Smith et al., 2009; Biswal et al., 2010; Khullar et al., 2011; Cetin et al., 2014). Another study (Mennes et al., 2010) proved the existence of spatial associations between brain regions that represent intrinsic dynamics and active during a cognitive task. Hence, increased interest to building the *functional connectome* with resting data has a vital role to build novel methods (Biswal et al., 2010; Allen et al., 2011a).

Co-existence of networks which are identified from resting brain data have been previously discussed (Calhoun et al., 2001; Smith et al., 2009; Allen et al., 2011a). Also, multiple networks exhibit temporal and spatial modulation during cognitive task vs. rest which shows existence of common spatial excitation patterns between these identified networks (Calhoun et al., 2008). Previous work (Khullar et al., 2011) has shown that structural and functional data may not have direct one-to-one correspondence and functional activation patterns in a well-defined structural region can vary across subjects even for a well-defined functional task. Existence of these neural activity patterns in multiple networks motivates us to investigate multiple resting-state networks as a single fusion template for functional normalization for multi groups of subjects.

We extend the previous approach (Khullar et al., 2011) by co-registering the multi group of subjects (healthy control and schizophrenia patients) by utilizing multiple resting-state networks (not just one as in the original work) as a single fusion template for functional normalization as an additional pre-processing step in contrast to the existing convention that only uses structure as a reference. The proposed method called ICA-based multi-network fusion template for functional normalization or “ICA-*m*fNORM” delineates resting fMRI data into INs using ICA and utilizes them as “functional templates” (FT) to derive normalization parameters. We attempt to utilize the normalization parameters (set of non-linear basis functions) computed by using multiple resting-state networks as a single fusion template for re-aligning each subject's fMRI data corresponding to a cognitive task such as the auditory oddball design (AOD). For every subject, the new AOD data is normalized to the group according to variations in functional systems unique to that subject. In this paper we describe the initial steps toward using multiple resting-state networks as a single fusion template for functional normalization. A simple wavelet-based image fusion approach is presented in order to evaluate the feasibility of combining multiple functional networks. Furthermore, we discuss the advantages, limitations and future direction of such an approach.

## Materials and methods

### Participants

Subjects in this study consisted of 28 healthy control (HC) adults, and 27 chronic schizophrenia patients (SP), all of whom gave written, informed, IRB approved consent at Hartford Hospital, CT and were compensated for their participation. Schizophrenia was diagnosed according to the DSM-IV TR criteria on the basis of a structured clinical interview administered by a research nurse and review of the medical file (First et al., 1995). Patients were slightly older than controls (SP age = 39.7 ± 10.1; HC age = 36.5 ± 11.3), but the difference was not statistically significant (two sample *t*-test *p*-value: 0.27).

### Experimental design and task

All participants were scanned during both an auditory odd-ball task (two times, each lasting 8-min) and while resting (one time, lasting 5-min), eyes open visually fixating on a cross. The AOD task stimulates a subject with three kinds of sounds: “standard” stimuli (1000 Hz tones with probability *p* = 0.8), infrequent target stimuli (1200 Hz tones, *p* = 0.1) and infrequent novel stimuli (computer generated complex tones, *p* = 0.1). The auditory stimuli were presented to each participant by a computer stimulus presentation system called visual and audio presentation package (http://nrc-iol.org/vapp/) via insert earphones attached within a pair of 30-dB noise-canceling MR compatible headphones. Stimuli were presented sequentially in pseudo-random order for 200 ms each with inter-stimulus interval varying randomly from 500 to 2000 ms across trials, with a mean of 1200 ms. In order to enable the participants to decipher stimulus tones from scanner noise, all stimuli were presented about 80 dB above the standard threshold of hearing. Each participant performed a practice block of 10 trials prior to entering the scanner. All participants reported that they could hear the stimuli and discriminate them from the background scanner noise. The participants were instructed to respond as quickly and accurately as possible with their right index fingers every time they heard the target stimulus (1000 Hz) and not respond at all if they heard any of the other two tones (standard or novel). The behavioral responses were recorded using an MRI compatible fiber-optic response device (Light-wave Medical, Vancouver, BC). The resting state scans were acquired while the participants rested quietly (with their eyes open) for 5 min without falling asleep inside the scanner. A detailed description of the AOD stimulus paradigm, the data acquisition techniques and previously found stimulus-related activation can be found in relevant work by Kiehl et al. (2005).

### Data acquisition

All scans were acquired at the Olin Neuropsychiatry Research Center at the Institute of Living/Hartford Hospital. A Siemens Allegra 3T MR system, equipped with 40 mT/m gradients and a standard quadrature head coil was used for all data collection. The scan started automatically by a trigger from the task paradigm controller. Following parameters were set for acquiring the functional scans trans-axially with gradient-echo EPI: repeat time (TR) = 1.50 s, echo time (TE) = 27 ms, field of view = 24 cm, acquisition matrix = 64 × 64, flip angle = 70°, voxel size = 3.75 × 3.75 × 4 mm, slice thickness = 4 mm, gap = 1 mm, 29 slices, ascending acquisition. The task fMRI data had 249 volumes in each run after discarding the 6 initial scans to compensate for longitudinal equilibrium (Calhoun et al., 2008). There were two back-to-back, separate runs for the task, and the data from the two runs were concatenated, resulting in 498 volumes.

### Data pre-processing

The magnitude of fMRI images (initially recorded as real and imaginary parts separately) were estimated and written as 4-D NIfTI (Neuroimaging Informatics Technology Initiative) files for further analysis. The standard pre-processing steps (realignment/motion correction and normalization) were performed via the SPM5 package (http://www.fil.ion.ucl.ac.uk/spm/software/spm5). The data were (a) motion corrected using an approach which minimizes the impact of local signal variations using the INRI align algorithm (Freire et al., 2002); (b) spatially normalized (Ashburner et al., 2000) into the MNI space using the EPI template provided with SPM5; and (c) slightly re-sampled (bi-linear interpolation) from 3.75 × 3.75 × 4 mm to a voxel size of 3 × 3 × 3 mm resulting in 53 × 63 × 46 voxels per volume. Conventionally, the last step is to spatially smooth the data using a full width half-maximum Gaussian kernel (10 × 10 × 10 mm).

### Group independent component analysis (GICA)

In order to derive FT, we used the GIFT Toolbox (http://mialab.mrn.org/software/gift/) and infomax algorithm for GICA (Bell and Sejnowski, 1995). We performed a subject-specific data reduction principal component analysis retaining 100 principal components (PC) using a standard economy size decomposition (Allen et al., 2011b). The relatively large number of subject-specific PCs has been shown to stabilize subsequent back-reconstruction (Erhardt et al., 2011b). In order to use memory more efficiently, further group data reduction was performed using expectation maximization (EM) principle component analysis algorithm (Roweis, 1998) and 20 PCs were retained.

We used a relatively low model order ICA (number of components, *C* = 20), since such models yield refined components that correspond to known anatomical and functional segmentation (Kiviniemi et al., 2009; Abou-Elseoud et al., 2010), though the approach we propose can be utilized for other model orders just as well. In order to estimate the reliability of the decomposition (Himberg et al., 2004), the Infomax ICA algorithm was applied repeatedly in ICASSO (http://research.ics.aalto.fi/ica/icasso/) and resulting components were clustered. Note also that the typical infomax algorithm used is actually jointly optimizing for both independence and sparsity (Calhoun et al., 2013).

### Pre-processing through co-design

Despite some interesting findings showing co-existing networks induced during rest and task (Calhoun et al., 2008; Smith et al., 2009), there has been little research in using these two data sets together. Mennes et al. (2010) demonstrated the possibility of predicting task-induced BOLD activity using inter-individual differences from resting-state networks. For advancing applications of these well-established and tested INs, we proposed the ICA-mfNORM framework and presented arguments for its application and position in the fMRI pre-processing pipeline by illustrating the benefits as well some methodological limitations for group-fMRI analysis. The increasing interest in new fMRI analysis methods developed using data-driven techniques such as ICA and multi-modal fusion tasks for re-defining fundamental questions posed prior to designing an fMRI study. Well-targeted and adaptive fMRI processing methods can help reveal hidden relationships across different networks of the brain. In subsequent sections we provide more details on our proposed approach.

#### Selection of relevant intrinsic networks

The auditory oddball task is known to induce BOLD signal increases in the temporal lobe network (Kiehl et al., 2005; Calhoun et al., 2008). The connectivity patterns in temporal regions within resting-fMRI data was demonstrated by Biswal et al. (1995), Greicius et al. (2003) and Kiviniemi et al. (2003). The consistency of the existence of these networks has been well-established through a group-ICA study of healthy subjects (Damoiseaux et al., 2006) followed by continuous experiments involving associations of these networks with covariates such as age, gender, and ethnicity (Allen et al., 2011a).

For our experiment, we depend upon the findings of numerous studies mentioned here, that the, selection of relevant INs is done based on two criteria's: (1) Known networks that are essential to brain function and comprise a robust array of components from previous studies such as occipital, sensory motor, parietal, inferior frontal, cingulate cortex and temporal regions. (2) The primary task-relevant region known from a data-set of interest, for e.g., temporal lobe and somatosensory motor in case of an auditory oddball task (AOD).

**Figure 1:**
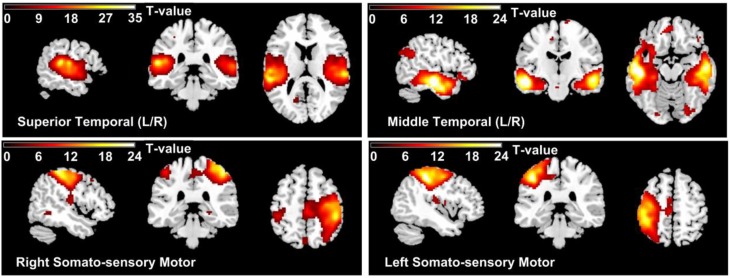
**Thresholded group mean spatial maps of relevant networks (1) Superior Temporal (2) Middle Temporal, (L/R), (3) Left Sensorimotor, and (4) Right Sensorimotor**.

Four INs are utilized to form the multi network template. These selected networks that are known to be positively modulated by the cognitive task—(1) superior temporal and (2) middle temporal, (L/R), (3) sensorimotor (L/R), (illustrated in Figure 1). They are actively modulated due to their known involvement (from other low-level experiments) with performing the task correctly. For example, the temporal lobe is the auditory region, and sensori-motor is responsible for motor functions such as tapping a finger when the target tone is heard (Kiehl et al., 2005; Calhoun et al., 2008). These INs were chosen due to implicit reasons associated with the nature of the cognitive task (AOD) involved in our experiment. For rest of the paper “*relevant networks*” refers to superior temporal, middle temporal, and sensorimotor networks.

### Estimating normalization parameters

The normalization parameters obtained from two different normalizations steps (pre-processing and functional normalization) are representative of the differences between average activities of each group with respect to a particular functional system/network.

In our experiment, two normalizations steps performed for the ICA-mfNORM procedure. The first normalization step was applied in the pre-processing section. The regular SPM normalization algorithm was applied to all data sets in the pre-processing stage in order to map all the data to the MNI template provided within SPM to present meaningful overlays of BOLD activity. Global shape differences between the functional network's boundaries of each subject and that of the group were obtained by suing SPM's spatial normalization algorithm which computes the 12-parameter affine model and the non-linear basis (Friston et al., 1995; Ashburner and Friston, 1999). Default settings of SPM were used to estimate the 392 parameters to describe deformations in each direction. The basis functions were estimated using 3-D discrete cosine transform (DCT) and regularization was done using λ = 0.01. The non-linear registration was performed as 12 iterations and normalization parameters were stored.

**Figure 2:**
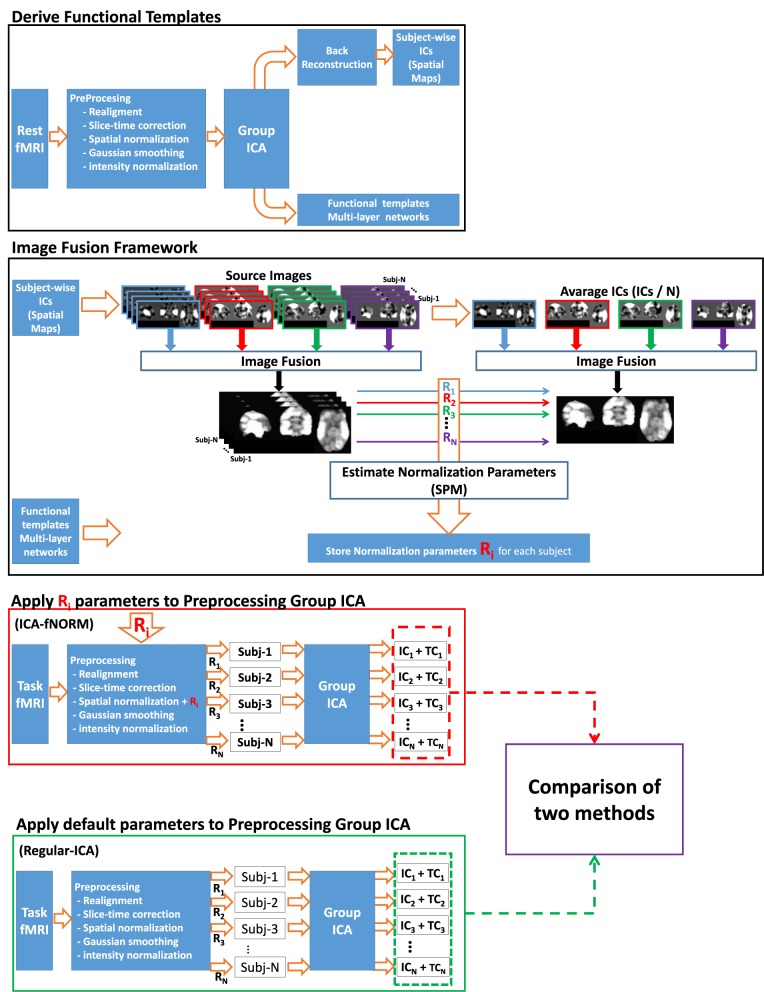
**Detailed flowchart of the proposed ICA-mfNORM framework illustrating all relevant stages involved**.

The second one was applied later in functional normalization. The normalization parameters that used for ICA-mfNORM (second stage normalization), for each subject were estimated using multi-network template as the reference image and that subject's multi-network components as the source image. The warping parameters (non-linear transformation ***R***) were computed for each subject (***R_i_:*** *Non-linear transformation of each subject*) and used for registration of the task related data corresponding to the same (*i*th) subject (see Figure 2).

### Multi-network fusion framework

Image fusion is an important and widely researched sub-area in the diverse field of image processing. In optics, imaging sensors can focus on certain objects in a single shot; this is controlled by the focal length of the lens used. Thus, to capture a focused representation of objects placed at different depths requires multiple images of the scene at various focal lengths followed by computational aggregation. This image set is commonly known as the focal stack. This aggregation process is achieved by different types of algorithms proposed in the literature, referred to as multi-focus image fusion. The advantages of using multi-focus data aggregated into a single image are manifold. The redundant information alongside complimentary features from various constituent images are much improved in a fused image. Other advantages are based on the position of image fusion in the image processing hierarchy, that is, several derivative applications like feature extraction, segmentation, compression take advantage of a good image fusion algorithm.

In the context of fMRI, the voxels contributing the most to the various networks derived from ICA have minimal spatial overlap. Resting-state data is decomposed into multiple components, thus providing enough flexibility to choose the appropriate networks that may correspond closely to a cognitive task that we are interested in studying. The functional template is then used in conjunction with the conventional spatial normalization process to adjust for inter-subject functional variability within the fMRI data associated with that cognitive-task.

**Figure 3:**
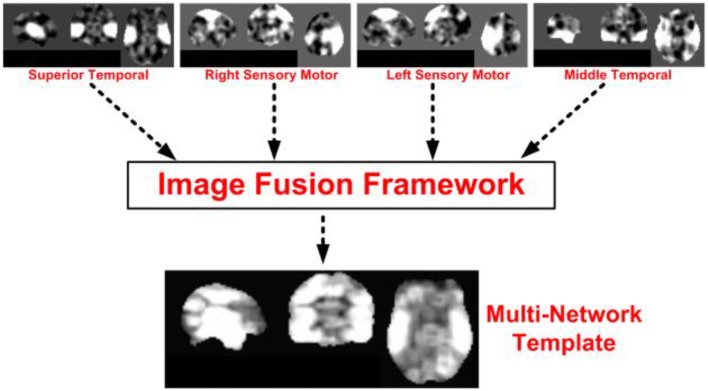
**An illustration of the proposed template formation framework from resting-state intrinsic networks**. The main functions of the image fusion block is to find an ideal combination of multiple networks, and provide priority values for a fused value for each voxel in the brain from multiple input networks.

The motivation for merging multiple INs is using resting-state networks as disjoint but aggregated representations of the functional organization of the brain. In order to better understand functional variability across individuals within a group or population, there is a need to account for the spatial characteristics of these functional networks. Thus, we propose a fusion methodology to merge multiple networks of functional organization into a single MNI-type functional template. An illustration of our proposed idea is presented in Figure 3.

Due to the complex nature of information embedded in these networks, merging them in to a single volumetric image is more challenging than just simple aggregation. As is the case with many template-based normalization algorithms, it is important to note that the method used to form the multi-network template may also depend on the registration methodology used.

#### Wavelets and image fusion

The most favored and established theory for image fusion, be it single or multi-sensor, is wavelet transforms. The majority of wavelet-based approaches utilize the discrete wavelet transform (DWT) where the wavelet coefficients of the source images are merged and the fused image reconstructed back to the original space using an inverse transform. However, the main limitation of this approach is that wavelets are unable to preserve the dynamic range of original data in the wavelet domain. This poses as a problem when dealing with fMRI networks which acquire arbitrary values as independent components. The second and more serious limitation in context to our application to fMRI is that the inverse transform is an approximation and may result in reconstruction errors. Such reconstruction errors are minimal when there is a single source image, and the application utilizes all the coefficients as is the case with image denoising. However, it is desirable to avoid reconstruction errors when multiple source images are combined based on a weighted coefficients or metric as generally practiced for image fusion.

Non-linear extensions of the DWT have been proposed through various schemes such as lifting (Sweldens, 1996) and morphological pyramids (Heijmans and Goutsias, 2000). The advantage of using non-linear extensions of the DWT for image fusion is manifold. It possesses many types of invariance properties: (1) Shift is achieved through the formulation of analysis (decomposition) and synthesis (reconstruction) operators, and (2) gray-value shift invariance provides a great advantage where gray values (activation intensities from different networks) are preserved in the fused image. That is, adding or multiplying a certain value throughout the volumetric image is equivalent to adding or multiplying by the same value during any step in the analysis (decomposition). The direct effect of this property is do with minimal or no change around the detail regions (edges, corners etc.).

For the purpose of demonstrating the possibility of using multi-network FT with our proposed ICA-mfNORM framework, we extend the multi-focal image fusion scheme presented by De and Chanda (2006) to fMRI analysis. We utilize the same scheme for the multi-network image fusion framework. There are two main reasons for choosing this particular methodology to achieve multi-network fusion: (1) This is a proof of concept strategy to demonstrate the feasibility of multi-network normalization, thus a simple to implement approach is adopted here. (2) This approach is capable of handling any number of INs as input to the fusion routine.

It is important to note that the proposed multi-network templates and ICA-mfNORM framework are in no way a replacement for the existing spatial normalization and registration schemes. Undermining the existing normalization approaches is not the goal of this work. The functional-template based normalization requires comprehensive validation on real fMRI data sets. However, the integration of various established approaches applied together in a single framework as ICA-mfNORM provide sufficient ground for experimenting with multi-network templates. The work presented in this paper will set a foundation for future studies that may apply ICA-mfNORM on a diverse variety of data sets and help validate it further. This type of progressive testing will eventually help bolster the position of such hybrid approaches that can possibly advance the field of fMRI analysis. The algorithm is divided in to three stages: (1) Analysis, (2) Fusion, and (3) Synthesis.

#### Analysis

**Figure 4:**
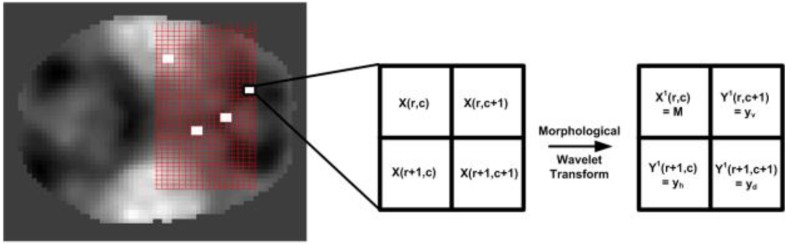
**An illustration of the subdivision of images in MN/4 matrices alongside decomposition of a 2 × 2 sub-matrix into approximation and detail bands**.

Unique analysis operators (forward wavelet transform)—(ψ^↑^, ω^↑^) and synthesis operators (reconstruction)—(ψ^↓^, ω^↓^) are used for a single level decomposition scheme. Due to the low resolution of the fMRI data, we restricted to a dual-resolution fusion scheme, which is only one level of decomposition for fusion. Further experimentation with multi-resolution may prove to be useful in analyzing performance. Let X is an image in a signal space V_0_which can further be decomposed in to subspaces V_1_ (approximation) and W_1_(detail) at level 1 in the wavelet domain. Let X ∈ V_0_ is a set of gray values in a 2-D space of size M × N such that M and N are both even. Thus, X can be subdivided in several disjoint 2 × 2 sub-images or blocks resulting in a total of MN/4 matrices as shown in Figure 4. Through quadrature down sampling, the analysis and synthesis operators ψ^↑^: V_0_ → V_1_ and ω^↑^: V_0_ → *W*_1_ are defined analytically in Equations (1, 2).

(1) $$\begin{array}{l} \psi^{↑}(X)(B)=M=max\{X(r,\;c),\;X(r,\;c\;+\;1),\;X(r+1,\;c),\\ \;X\text{(}r\text{ + 1, }c\text{ + 1)\},} \end{array}$$

(2) $$\omega^{↑}(X)(B)\;=(y_{v},\;y_{h},\;y_{d}).$$

where *y_v_, y_h_, y_d_* represent the vertical, horizontal and diagonal detail signals, respectively and are defined as:

(3) $$y_{v}\;=\{\begin{array}{c} M-X\left(r,\;c\;+\;1\right)\;if\;M-X(r,\;c\;+\;1)\;>\;0,\\ X(r,\;c\;+\;1)-M\;otherwise, \end{array}$$

(4) $$y_{h}\;=\{\begin{array}{c} M-X\left(r\;+\;1,\;c\right)\;if\;M-X(r\;+\;1,\;c)\;>\;0,\\ X(r\;+\;1,\;c)-M\;otherwise, \end{array}$$

(5) $$y_{d}\;=\{\begin{array}{c} M-X\left(r\;+\;1,\;c\;+\;1\right)\;if\;M-X(r\;+\;1,\;c\;+\;1)\;>\;0,\\ X(r\;+\;1,\;c\;+\;1)-M\;otherwise, \end{array}$$

**Figure 5:**
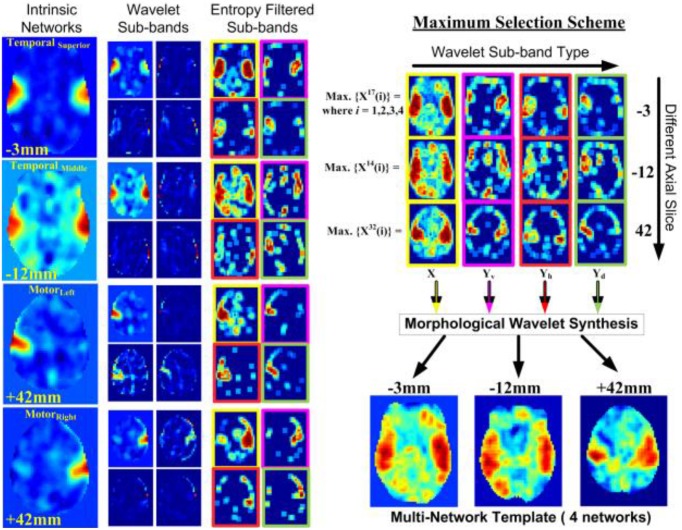
**MWT Example using Intrinsic Networks; An example showing step-by-step illustration of different stages of the proposed entropy-filtering based wavelet fusion scheme**. The final image is obtained through the stacked-image maximum selection scheme in the wavelet domain followed by morphological reconstruction. An orthogonal view (arbitrarily chosen) of the resulting template is illustrated depicting active regions present in the four input templates.

The scaled approximation (M in Equation 1) and detail (Equations 3–4) values is, approximation X^1^ and details Y^1^, respectively. Thus, these 4 values can be stored in a 2 × 2 matrix as shown in Figure 4. The above step is repeated for *n* different INs (chosen by the user) that are given by X_1_, X_2_, X_2_,., X_n_, thus resulting in a pair of coefficient matrices for each network—X = X_i_, Y_i_. An example illustrating the above wavelet synthesis on an axial slice from the target resting-state networks used for fusion are shown in Figure 5 (see second column). For clarity, we select 3 different axial slices (at −3, −12, and +42 mm in MNI coordinate space), each corresponding to the maximum value in at least one network. This illustrates the intensity distribution across various networks that exist as a volumetric image.

An important point to note in Figure 5 is the range of transformed images. If all values of the image X belong to the range [0, R], then the transformed approximation signal X belongs to the range [0, R] whereas the detail signal Y belongs to the range [−R, R]. Larger values in any image X_i_ corresponds to a brighter pixel whereas a large value in Y_i_ corresponds to a possible edge, corner or other high-spatial frequency features.

#### Fusion

After the completion of analysis operation, 2 sets of *n approximation* (*X^i^*_1_) and *detail* (*Y^i^*_1_) signals are obtained. Based on the range of these images as discussed above, apriority condition is required to choose the network that may contribute to the pixel value at each location (*r, c*). In other words, the priority condition is basically the fundamental fusion criterion to merge multiple target images, INs in this case. There are several fusion criteria proposed in the literature as listed below:

- *Maximum selection (MS) scheme*: This scheme simply picks the coefficient from each sub-band which has the largest magnitude across multiple images.
- *Weighted average (WA) scheme*: This technique was introduced by Burt and Kolczynski (1993) utilizes a neighborhood based normalized correlation among target images' sub-bands. The final fused coefficient is estimated through a weighted average of the two images' coefficients. The disadvantage of this method is that it cannot work with more than two images due to the application of the correlation operator.
- *Window-based verification (WBV) scheme:* This scheme is probably the most sophisticated amongst all and has been used for several applications in combination with some other techniques (Li et al., 1995). It performs a fusion by means of a binary decision map estimated over a local neighborhood for two or more coefficients from the target images. The binary decision map decides what target image contributes to a pixel (*r, c*) in the final fused image.

For our implementation, we utilize the MS scheme that is also utilized by De and Chanda (2006) for fusion of multi-focus images. A minor, but substantial novel contribution to the MS scheme for fusion is proposed here. A pre-processing step to obtain a crude segmentation of meaningful voxels within the sub-bands is performed using an entropy filtering approach. Each voxel value is replaced by its entropy value (a statistical measure of randomness) in the neighboring 3 × 3 neighborhood. It is computed as: *H*(*x*) = ∑*_N_P_N_*(*x*)log_2_ (*P_N_*(*x*)), where (*x*) is the center pixel and *P_N_*(*x*) is the probability distribution (histogram) in neighborhood *N*. As noticed from Figure 5, the difference between the regularly processed sub-bands and the entropy-filtered bands is quite significant. The regions with higher activation values are now more prevalent in the filtered images (third column). Some of the voxels with low local entropy are removed whereas the activation specific voxels are scaled based on their local entropy. This assists in image fusion where the activation across different networks is spread over a number of voxels in different planes (axial slices here) across the brain. This also serves as the primary reason for demonstrating the fusion process with the help of three different slices extracted from four different INs −3 mm for superior temporal, −12 mm for middle temporal, and +42 mm.

An alternate approach that may utilize some type of functional connectivity metric to realize the weights of each component may be worthwhile to pursue. Here, we presented a proof of concept for functional template formation. Experimenting with fusion methods is outside the scope of this work but considered to be a substantially important part of future work.

The MS fusion scheme compares multiple INs {*X_i_, i* = 1, 2,.*n*} and combines them into **X** = {*X*^1^*_f_, Y*^1^_*f*_}, where *X*^1^_*f*_ and *Y*^1^_*f*_ are given by *X*^1^_*f*_(*r, c*) = *max*{|*X*_1_(*r, c*)|,|*X*_2_(*r, c*)|,.|*X_n_*(*r, c*)|} and *Y*^1^_*f*_(*r, c*)= max{|*Y*_1_(*r, c*)|,|*Y*_2_(*r, c*)|,.|*Y_n_*(*r, c*)|}, respectively. This operation results in 4 fused images (1 approximation, and 3 details) consisting of features from all the networks. These results are presented in Figure 5 that shows initial images and the fused result using 4 different networks for different slices and sub-bands.

#### Synthesis

The last and final step in template formation is the synthesis or reconstruction back from wavelet domain V_1_ to the signal domain V_0_. The fused image X ∈ V_0_ is reconstructed by applying the synthesis operators ψ^↓^ and ω^↓^ on the transformed and fused set **X** = {*X*^1^_*f*_, *Y*^1^_*f*_}, The reconstruction to obtain the synthesized (fused) signal is done through the following equations:

(6) $$\begin{array}{l} \hat{X}(r,\;c)=\hat{X}(r,\;c+1)=\hat{X}(r+1,\;c)=\hat{X}(r+1,\;c+1)\\ \text{ = }M \end{array}$$

(7) $$\begin{array}{ccc} \hat{Y}(r,\;c) & = & \text{min}(y_{v},y_{h},y_{d},0),\\ \hat{Y}(r,\;c+1) & \text{=} & \text{min}(-y_{v},0),\\ \hat{Y}(r+1,\;c) & \text{=} & \text{min}(-y_{h},0),\\ \hat{Y}(r+1,\;c+1) & = & \text{min}(-y_{d},0), \end{array}$$

**Figure 6:**
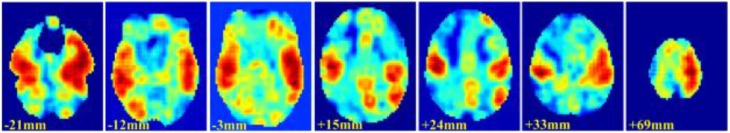
**Illustration of the multi-network functional template through a few selected slices spread throughout the brain**. This image highlights the merging of features from various networks, also shown in Figure 5.

The reconstructed signal X‘ at any point (*u, v*) ∈ {(*r, c*), (*r, c* + 1), (*r* + 1, *c*), (*r* + 1, *c* + 1)} is given by Equation (8) and illustrated in Figure 6 through various slices.

(8) $$X‘(u,\;v)=\hat{X}(u,\;v)+\hat{Y}(u,\;v)$$

The final fused image is taken as the functional-template and used in conjunction with the ICA-mfNORM frame work as presented in the Figure 2. In regard to the importance given to performance analysis for fMRI pre-processing algorithms, we present the results of the fusion algorithm proposed above and compare it with regular ICA analysis.

## Results

The multi-network functional template is developed using manually relevant INs that are known to be positively modulated by the cognitive task—superior temporal, middle temporal, and sensorimotor networks (Biswal et al., 1995; Greicius et al., 2003; Kiviniemi et al., 2003; Kiehl et al., 2005; Calhoun et al., 2008; Khullar et al., 2011). All these networks are actively modulated due to their known involvement (from other low-level experiments) with performing the task correctly. For example, the temporal lobe is the auditory region, and sensorimotor is responsible for motor functions such as tapping a finger when the target tone is heard.

In order to obtain an initial estimate of the advantages of a multi-network functional normalization approach, we examined some analysis' such as; comparison of the component maps to obtain the number of active voxels and evaluate the local correlation performance, investigating the multi-group comparison to obtain group differences and running two different classification algorithms to examine the improvement for classification of the AOD group data containing 28 HCs and 27 SPs before and after ICA-mfNORM.

### The activation maps

#### Single group comparison; one-sample t-test

The results of the single group comparison for the group level *t*-maps are presented in Table 1. The activation maps corresponding to the primary task-positive component from functionally realigned AOD data were analyzed and the most significant voxels were derived. The activation maps were analyzed using the automatic anatomical labeling (AAL) atlas (Tzourio-Mazoyer et al., 2002) that contains several different regions to parcellate the functional activation maps anatomically of most significant voxels. One-sample *t*-tests were performed on networks of interest and the activation maps were corrected for multiple comparisons (*p* < 0.01) by using the false discovery rate (FDR) method (Chumbley and Friston, 2009). For all regions labeled within the threshold *t*-maps for with and without ICA-mfNORM methods, the location of maxima (*x, y, z*), mean *t*-value and number of significant voxels above the threshold were computed. Then spatial shifts inactivation foci between the two approaches were computed as the Euclidean distance (ED) between the local maxima.

**Table 1:** **Activation maps comparison for Regular-ICA and ICA-mfNORM**.

**Anatomical Regions**	**Regular—ICA**					**ICA-mfNORM**					**% Gain**		
	**Coordinates (x,y,z)**			**tm**	**Vol (*p* < 0.01)**	**Coordinates (x,y,z)**			**tm**	**Vol (*p* < 0.01)**	**Local**	**Network**	**ED (mm)**
L superior temporal	60	21	9	6.16	1349	60	21	9	6.16	1615	19.72	14.74	0.00
R superior temporal	−63	18	9	5.86	1296	−63	18	6	5.98	1420	9.57		3.00
L middle temporal	51	30	51	5.36	1229	54	24	42	5.40	1282	4.31	3.21	11.22
R middle temporal	0	−9	45	5.40	956	0	−9	42	6.03	883	−7.64		3.00
Right post-central	−60	15	39	4.76	524	−63	18	33	5.06	631	20.42		7.35
L sensorimotor	45	−15	−12	8.01	749	42	−12	−18	8.61	826	10.28	5.85	7.35
R sensorimotor	−48	−15	−12	7.80	789	−45	−12	−18	7.99	802	1.65		7.35

*The results of the ROI analysis for the group level t-maps*.

We identified six anatomical regions, left superior temporal, right superior temporal, left middle temporal, right middle temporal, left sensorimotor, right sensorimotor. All regions showed positive gain and similar patterns were observed in volume calculation (*p* < 0.01). Significant improvements in local maxima were observed for all regions see Table 1. In addition to these regions, inferior temporal regions and thalamus showed increased activation patterns after ICA-mfNORM.

#### Local correlation performance of the ICA-mfNORM

We also evaluated the local correlation performance of relevant networks. The effect of ICA-mfNORM was evaluated based on correlation of the subject specific relevant networks to corresponding mean relevant networks between subjects obtained by applying ICA with and without ICA-mfNORM by holding other settings as described before.

Correlation of the subject specific relevant networks components obtained by applying ICA with ICA-mfNORM showed higher correlation except for sensorimotor regions, which showed a small loss. For subject specific relevant networks, we used paired *t*-tests on the correlation results. The cut off *p*-value for all of the tests is set at *p* < 0.05. The results did not show significant improvement for local correlation performance of relevant networks. Table 2 shows average correlation of each relevant network to corresponding mean correlation of the relevant networks between subjects with and without ICA-mfNORM.

**Table 2:** **Average correlation of the each subject specific relevant networks to corresponding mean relevant networks between subjects with and without ICA-mfNORM**.

**Anatomical Regions**	**Reg-ICA**	**ICA-mfNORM**		
	**Corr (Std)**	**Corr (Std)**	***p*-value**	**% gain**
Superior temporal	0.532 (0.13)	0.57 (0.12)	0.1677	7.14
Middle temporal	0.53 (0.11)	0.56 (0.11)	0.3331	5.66
Sensorimotor	0.502 (0.1)	0.495 (0.11)	0.5145	−1.3

#### Comparison of normalization

In our experiment two normalizations steps were performed for the ICA-mfNORM procedure. The first one was applied in the pre-processing section and the second was applied later in functional normalization. This process might raise a concern regarding this concatenation of transforms. In order to address this concern, instead of using the functional template in the second step, we used an average of the first timepoint of the EPI data from the already normalized subjects. This method is widely used to produce a “*study specific template*.” Then we compared two approaches, both of which use two transformations, but only one (ICA-mfNORM) uses the functional information.

**Figure 7:**
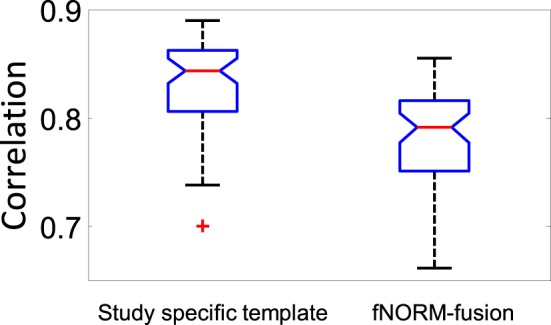
**Correlation comparison of normalization for study-specific template and ICA-mfNORM. +: outliers for +/− 2.7σ**.

We created a template image from the average of the first image across all subject then we computed the correlation scores of the template image with the first image across all subject. This process was repeated for both the study-specific template and ICA-mfNORM; we then compared the correlation results see Figure 7. Individual subjects showed greater spatial correlation to the mean for the study specific template approach as we optimized the similarity of structure. Then we performed GICA on data that was normalized to the study-specific template and compared one-sample t-maps of relevant networks (Superior Temporal, Middle Temporal, Sensorimotor) to those obtained using ICA-mfNORM. ICA-mfNORM showed higher activation (*t*-value) than the study-specific template for these networks. This suggests the two-step process alone is not causing the performance improvements, but rather the introduction of functional information is the main difference. Results are displayed in Table 3.

**Table 3:** **Activation maps comparison for study-specific template and ICA-mfNORM**.

**Anatomical Regions**	**Study specific template**					**ICA-mfNORM**					**% gain**		
	**Coordinates**			**tm**	**Vol (*p* < 0.01)**	**Coordinates**			**tm**	**Vol (*p* < 0.01)**	**Local**	**Network**	**ED (mm)**
	**(x,y,z)**					**(x,y,z)**							
L superior temporal	63	21	9	6.53	1450	60	21	9	6.27	1615	11.38	25.72	3.00
R superior temporal	−63	18	9	6.39	964	−63	18	6	5.98	1420	47.30		3.00
L middle temporal	45	15	42	4.41	231	54	24	42	5.40	1282	454.98	98.44	12.73
R middle temporal	0	−15	45	7.30	1051	0	−9	42	6.03	883	−15.98		6.71
Right post-central	−66	24	27	6.08	127	−63	18	33	5.06	631	396.85		9.00
L sensorimotor	42	−12	−18	10.66	438	42	−12	−18	8.61	826	88.58	79.30	0.00
R sensorimotor	−45	−9	−18	10.70	470	−45	−12	−18	7.99	802	70.64		3.00

*The results of the ROI analysis for the group level t-maps*.

### Multi-group comparison—functional network correlation

Functional network connectivity (FNC) is a correlation value that summarize the overall connection between independent brain maps over time (Jafri et al., 2008; Arbabshirani et al., 2013). Therefore, the FNC feature gives a picture of the connectivity pattern over time between independent components. The provided FNC information was obtained from fMRI from a set of SPs and HCs at rest, using GICA. The GICA decomposition of the pre-processed fMRI data resulted in a set of brain maps, and corresponding timecourses. These timecourses indicated the activity level of the corresponding brain map at each point in time. The FNC features were the pair-wise correlations between these timecourses, for each subject. FNC indicates a subject's overall level of “synchronicity” between brain areas. Because this information is derived from fMRI scans, FNCs are considered a functional modality feature (i.e., they describe patterns of the brain function).

**Figure 8:**
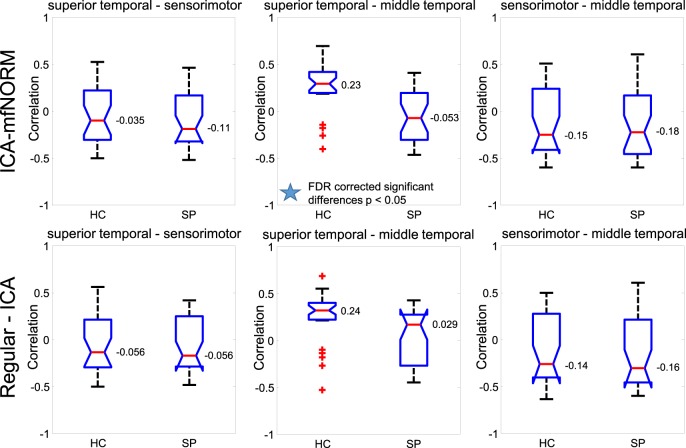
**Functional Network Connectivity with and without ICA-mfNORM for HC, SP, and FDR corrected differences between the groups for Superior Temporal, Sensorimotor, Middle Temporal. +: outliers for +/− 2.7σ**.

For the purpose of finding FNC differences between HCs and SPs groups through the AOD task, we were primarily interested in analyzing the correlation of temporal lobe component with others (Kiehl et al., 2005; Calhoun et al., 2008) as these are strongly modulated by the cognitive task. The FNC results for the average of each group and the mean correlation difference between HCs and SPs was computed for each relevant network pairs results are shown in Figure 8. To determine, which correlation pairs were significantly different between HC and SP two-sample *t*-tests were performed. The cut off *p*-value for all of the tests is set at *p* < 0.05 and was corrected for multiple comparisons using the FDR method. In our experiment correlation of superior temporal and middle temporal regions showed significant differences between the HCs and SPs for the data obtained with ICA-mfNORM method while there is no significant difference for the data obtained from regular-ICA method. Also, in order to investigate the significant interaction between regular-ICA and ICA-mfNORM, we used Two-Way ANOVA, results showed significant differences at *p* < 0.05 between these two modalities for superior temporal and middle temporal regions. Other relevant network pairs did not show any significant differences.

### Classification comparison

Activation topography within the temporal lobe network has repeatedly been used as a basis of classification in HCs and SPs showing high accuracy and robust activation patterns (Calhoun et al., 2004). We validated our method using a cross-validation strategy based on the two different types of classification algorithms which are linear discriminant classifier and shapelet based classification. All types of data are obtained by applying ICA with and without ICA-mfNORM by holding other settings as described before. The same training and testing sets are used for all methods (with ICA-mfNORM and without ICA-mfNORM) for each run.

#### Classification with functional network correlation

To examine the effect of ICA-mfNORM to classification, FNC scores were obtained by applying ICA with and without ICA-mfNORM by holding other settings as described before. FNC scores of relevant networks (3 scores for each subject) were used as features for classification. FNC scores of relevant networks for 14 HC and 14 SP subjects were used for training set. FNC scores of relevant networks for 14 HC and 13 SP subjects were used for testing set. The classifier repeated 100 runs and for each run train and test sets were assigned randomly.

We used a relatively simple classifier, the linear discriminant classifier (Duda et al., 2001), to evaluate the effect of ICA-mfNORM by isolating the effect of advanced classification algorithms. The average accuracy of the classifier for FNC scores which was obtained by applying ICA without ICA-mfNORM is 56.6% (std: 7.25) and with ICA-mfNORM is 64% (std: 7.3). Paired *t*-tests on the accuracy results over 100 iterations demonstrated a significant improvement in classification (*p* < 0.005).

#### Classification with shapelets

**Figure 9:**
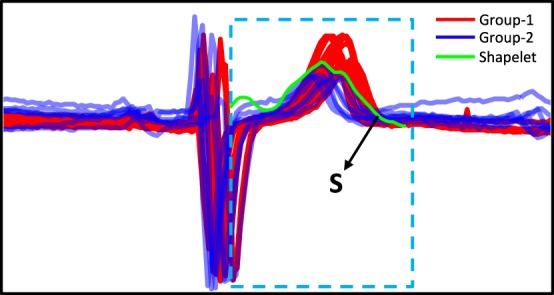
**An example of shapelet**. Heartbeats of a 67 year old male in two different days shown in blue and red. Red has a higher peak than the blue in the frame. The shapelet (S) that distinguishes the classes most (Cetin et al., 2015).

In order to evaluate the effect of ICA-mfNORM pre-processing for classification, we also used shapelet based classification (Rakthanmanon and Keogh, 2013; Cetin et al., 2015) as an alternative to linear discriminant classifier. This gave us a chance to evaluate the effect with a non-statistical method. Time series shapelets are small segments of time series that distinguish between classes based on existence of such segments in the classes. For simplicity and better understanding of shapelet algorithm, Figure 9 shows a shapelet example by using heartbeats (ECG) data of a 67 year old male in two different days shown in blue and red. Details of shapelet algorithm can be found in Cetin et al. (2015) and Rakthanmanon and Keogh (2013).

Time series of relevant network components were obtained by applying regular ICA and ICA-mfNORM. Time series of relevant network components for each subject are used as a time series domain for shapelet based classification. Time series of the relevant network components for 14 HC and 14 SP subjects are used for training data set. Time series of the relevant network components for rest of the subjects (14 HC and 13 SP) are used for testing data set. The shapelet algorithm finds a *shapelet* by using training data set and then assigns a label (HC or SP) for each subject in the testing set by using the *shapelet*. The entire process was repeated 100 times for relevant network components and the average accuracy of these scores was recorded as a final classification accuracy scores for each method and each components.

Finally, to determine the significant accuracy differences of ICA-mfNORM vs. regular-ICA, paired *t*-tests were conducted on the two groups (100 accuracy scores for each group). The cut-off *p*-value for all of the tests was set at *p* < 0.005. Results showed significant accuracy improvement as follows; accuracy for superior temporal is 71.96% (std: 5.25) with ICA-mfNORM and 57.97% (std: 4.45) without ICA-mfNORM, is 69.33% (std: 8.92) with ICA-mfNORM and 51.54% (std: 3.96) without ICA-mfNORM, middle temporal is 62.7% (std: 8.57) with ICA-mfNORM and 56.37% (std: 4.84) without ICA-mfNORM. Thus, classification results were considerably better with ICA-mfNORM compared to regular-ICA.

## Discussion

In this study, we present the initial exploratory effort in a new and upcoming area of research in the fMRI community; a foundational methodology for forming multi-network FT that may be used for functional normalization or realignment. This paradigm shift has been inspired by the availability of large amounts of data as well as increasing interest in the resting-state function of the brain and its interaction with task-modulated networks.

We investigated the correlation of each relevant network components to corresponding mean relevant network components between subjects with and without ICA-mfNORM. Our results showed that the proposed ICA-mfNORM method obtained more correlated components. Also, we compared ICA-mfNORM template with study specific template to examine the effect of greater image smoothness. Our results demonstrated that ICA-mfNORM showed higher activation (*t*-value) and extent (cluster size) than study-specific template for relevant networks.

The results of the single group comparison for the group level *t*-maps provided evidence showing how proposed ICA-mfNORM, using functional information from selected INs helped improve various aspects of post-analysis results such as positive gain in volume calculation, *t*-statistics and detection sensitivity and possibly help identify differences in region wise activity that may go unnoticed otherwise such as increased activation patterns of inferior temporal regions and thalamus. One of the interesting sites of activation clusters that appear after the fusion template is applied in the pre-processing stage is near the thalamus, which happens to be the communication hub of the brain where many networks converge. A higher activity in this region in patients may indicate hyperactivity patterns that may be closely connected with the activity in the temporal lobe region. Furthermore, thalamic abnormalities in schizophrenia are well-documented (Goff and Coyle, 2001; Woodward, 2012). Recent research suggests that these regions play a role related to production of auditory verbal hallucinations (Hoffman and Hampson, 2012) along with the putamen, which also showed a significant static thalamic connectivity effect.

Using the temporal lobe component to find the differences for HCs and SPs through the AOD task is studied by Kiehl et al. (2005) and Calhoun et al. (2008) as these are strongly modulated by the cognitive task. A multi-group comparison with functional network correlation showed significant differences between correlation of patients and controls within the temporal lobe components. The use of functional information relating to resting-state data in patients seems to play a larger role in uncovering hidden interactions within important regions of interest for a simple task as the AOD.

In current study, we also found higher group differences between the HCs and SPs with ICA-mfNORM method then without ICA-mfNORM method. Particularly, we found significant FNC differences involving elements of the superior temporal and middle temporal regions between the HCs and SPs for the data obtained with ICA-mfNORM method while there is no significant difference for the data obtained without ICA-mfNORM method. These group differences provide motivation for understanding how connectivity patterns differ in response to these different stimulus conditions. And we test our motivation with two different classification algorithms. The results showed that our method improved our ability to classify the groups. All of this information provides strong support for incorporation of methods that can improve functional registration among subjects.

### Limitations and future work

The application of FT is a relatively new area of research. As per our knowledge, it has not been proposed or used previously in fMRI analysis studies. Our method come attached certain limitations as with every new method, especially when the system of application is as complex as the human brain.

The combination of multiple INs has implications for how one interprets each of these temporally coherent networks on their own. That is, subtle effects of each network on the normalization methodology are difficult to separate and identify. Having said that, resting state networks are known to have complex interactions across the brain and it is important to carefully choose the robust and task-relevant networks for template fusion and functional normalization. Another point to note is that since the data collected during AOD contains task-related variance, it is difficult to know if the physiologic mechanism behind the patient vs. control changes is the same for both paradigms (rest and task). It may be possible that AOD changes are the mixture of the two effects (rest and task), and are only enhanced when resting-state networks are used to normalize the group data.

The future directions are 2-fold. Firstly, further validation and analysis of the proposed morphological wavelet-based methodology is required to understand the implications of using such a pick the best voxel type of approach as compared to using the model-driven approaches in the literature such as deriving mixture of probability distributions or so on. The analysis or comparison of other fusion methodologies is outside the scope of this study, but shall be an important part of the future work. Secondly, an exciting future direction is to be generating templates adaptively based on a chosen task. A large database of aggregated resting state networks similar to the one presented by Allen et al. (2011a) may play a large role in understanding the formulation of multi-network templates. A database where any researchers may choose relevant anatomical regions for their study and the system or algorithm presents with multiple templates formed using different combinations of resting state networks that correspond to function in the regions mentioned by the researcher. This may well become a crowd-sourced testing methodology for this method, as it is best to validate the method using large number of applied studies due to the different types of variability (demographic, health, task etc.) associated with fMRI data sets collected and analyzed across the functional brain imaging community. In this study, we limit the work by co-registering multi group of subjects (HC and SP) and by utilizing their relevant networks that are active during task and rest state (Calhoun et al., 2008; Smith et al., 2009) to demonstrate the effectiveness and a proof-of-concept in context to our approach. However, in the future we plan to extend this to incorporate all the INs being used together. We also used a two-step process in this initial work, whereas future work will focus on a joint function/structure co-registration in a single step.

## Conclusions

Our results provides evidence that combining multiple INs into a single functional template, and using this template to incorporate functional information into the spatial normalization process both improve the task activation and our sensitivity to group differences. Such a result suggests also that functional boundaries, known to vary considerably across individuals, may vary in a systematic way in SP vs. HC. Results thus strongly demonstrate the importance of characterizing the spatial location of functional domains within individuals prior to a group analysis.

### Conflict of interest statement

The authors declare that the research was conducted in the absence of any commercial or financial relationships that could be construed as a potential conflict of interest.
